# p‑Type Surface Defects on n‑GaN Nanorods

**DOI:** 10.1021/acs.nanolett.5c01839

**Published:** 2025-05-19

**Authors:** Sumesh Sadhujan, Sherina Harilal, Kefan Zhang, Riam Abu Much, Abdullah AbuBekr, Ayat Asleh, Awad Shalabny, Amro Sweedan, Nursidik Yulianto, Andam Deatama Refino, Hutomo Suryo Wasisto, Laila Abu Madegam, Aeid Igbaria, Mariela J. Pavan, Muhammad Y. Bashouti

**Affiliations:** ◧ Department of Solar Energy and Environmental Physics, Swiss Institute for Dryland Environmental and Energy Research, J. Blaustein Institutes for Desert Research, 26732 Ben-Gurion University of the Negev, Midreshset Ben-Gurion, Building 26, 8499000, Israel; ‡ The Academic Arab College for Education in Israel-Haifa, 22 Hahashmal St., Haifa 33145, Israel; § The Ilse-Katz Institute for Nanoscale Science & Technology, Ben-Gurion University of the Negev, POB 653, Marcus Family Campus, Building 51, Beer-Sheva 8410501, Israel; ∥ Institute of Semiconductor Technology (IHT) and Laboratory for Emerging Nanometrology (LENA), 26527 Technische Universität Braunschweig, Braunschweig 38106, Germany; ⊥ Research Center for Photonics, National Research and Innovation Agency (BRIN), Kawasan Puspiptek Gd. 442, South Tangerang 15314, Indonesia; # Engineering Physics Program, Institut Teknologi Sumatera (ITERA), Jl. Terusan Ryacudu, Way Huwi, Lampung Selatan, Lampung 35365, Indonesia; ○ PT Nanosense Instrument Indonesia, Umbulharjo, Yogyakarta 55167, Indonesia; ● The Department of Life Sciences, Ben Gurion University, Beersheba 841050, Israel

**Keywords:** GaN, Surface doping, Surface states, Surface photovoltage inversion, Electrostatic field

## Abstract

Nanowire surfaces are of particular interest, primarily for their potential in optoelectronic applications. Thus, different surface treatments have been performed to develop methods for controlling the surface effect. Here, we successfully shifted the n-type surface states in n-type GaN nanorods to p-type states in the challenging regime, i.e., the blue regime. This was achieved through reverse charge transfer driven by an electrostatic field induced by surface strain. The p-type surface state demonstrates an inverted photovoltage mechanism, as well as a stable blue photoluminescence at room temperature under ambient conditions, within the n-type GaN nanorods. The inverted charge transfer at the surface of the GaN nanorod array was determined by X-ray photoelectron spectroscopy (XPS), surface photovoltage, Kelvin probe, Raman, and photoluminescence measurements. The mechanism and the study’s conclusions have been supported experimentally and theoretically. This surface state inversion approach offers a new strategy for regulating p–n junctions in low-dimensional nanomaterials.

## Graphical abstract

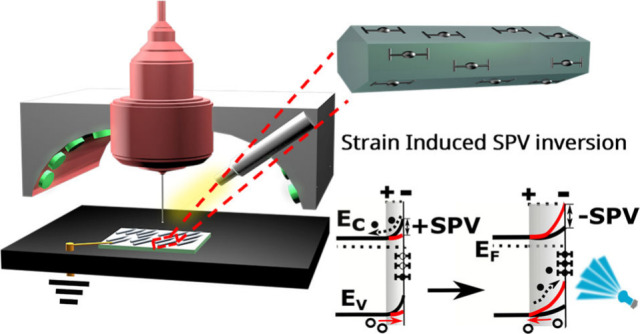

Artificial sources of light have dramatically transformed our society through a variety of applications in areas such as communication, transportation, healthcare, and others.^1–3^ Since lighting accounts for approximately 20–30% of global energy consumption, energy-saving solutions, such as light-emitting diodes (LEDs), have become essential.^3–7^ For example, LEDs based on gallium nitride (GaN) have already achieved certified power savings, as they require 10 times less energy than ordinary light bulbs.^3–7^ To obtain a p–n junction for GaN-LEDs, a junction between an n-type GaN and a p-type GaN must be developed. It is easier to create the n-type GaN with high electron carrier concentrations (the majority of carriers are electrons, i.e., n̅) of 10^18^–10^19^ cm^–3^ due to the nitrogen vacancies.^8,9^ However, it is challenging to fabricate GaN with high concentrations of holes (i.e., p̅), and it took several decades to realize the p-type GaN in the bulk regime. This was accomplished mainly through Mg doping.^10–14^ In addition, at the nano/microscale, Mg doping has limited solubility in GaN. Less than 1% of Mg forms acceptor centers at room temperature due to its high activation energy (approximately ≈ 200 meV).^15–17^ Hence, there is considerable interest in fabricating GaN with a p-type character, which would be useful in nano/microlevel optoelectronic device applications.

To this end, we obtained shallow defects with a p-type character in n-type GaN nanorods (GaN NRs) in the m-plane through an etching process, which avoids the above-mentioned material problems, inverts the majority carrier concentration (from n to p), and generates a p–n junction in the challenging regime, i.e., the blue regime. The shallow p-type defects demonstrate stable blue photoluminescence at room temperature under ambient conditions and illustrate an inverted photovoltage mechanism. Generally, one always strives to neutralize surface defects in the semiconductor industry. Indeed, defects can impede charge transport by trapping or scattering. However, as in our case, they can also boost charge transport by generating extra free-charge carriers through surface strain.^18–21^ This effect of surface defects is intensified in the nanoregime, as they strongly influence the device’s performance due to the increased surface-to-volume ratio.^22^

In this study, we demonstrated the p-type defects on n-type GaN NRs and their role in semiconductor optoelectronic properties. This was achieved by combining nondestructive optoelectronic emission spectroscopies based on super- (i.e., hυ > E_g_) and sub-bandgap (i.e., hυ < E_g_) illumination (Figure 1). Specifically, we developed *in situ* measurements incorporating the Kelvin Probe (KP), surface photovoltage (SPV) and surface photovoltage spectroscopy (SPS), done on the same spot to eliminate errors and to precisely probe the defect properties.^23^ The study was also supported by X-ray photoelectron spectroscopy (XPS). Additionally, we introduced the hydroxyl (HO^–^) group onto the GaN surface as an effective applied method to specify the role of the p-type defect in the stable photoluminescence process. The success of the chemical etching approach helped us to resolve not only the origin of the defects but also the intercharge transfer between defects and bands. Addressing the surface defect in wide-bandgap semiconductors could shed light on optoelectronic descriptors for applications in low-dimensional nanomaterials for cost-effective energy technologies.^24,25^ The shallow p-type defect facilitates efficient charge transfer between bands and defects, which can translate into a low-power device operation, a crucial energy savings component.^26^

**Figure 1:**
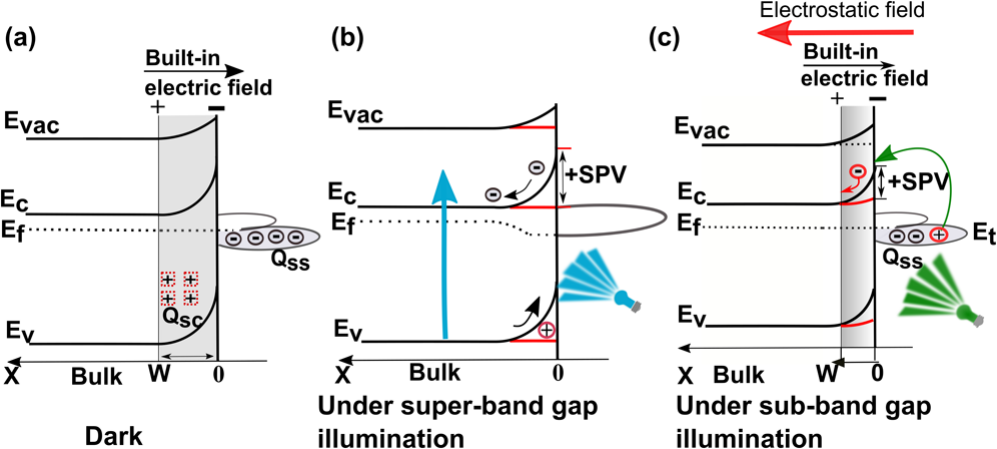
(a) Band edge in thermal equilibrium. Transition process involved in (b) super-bandgap illumination hυ > E_g_, and (c) sub-bandgap illumination surface state (Q_ss_) depopulation by hυ < E_g_.

The GaN NRs were realized using the inductively coupled plasma reactiveion etching (ICP-RIE) method on a ∼4.8 μm-thick GaN bulk layer grown by metal-organic vapor phase epitaxy (MOVPE), doped with silicon (10^17^ cm^–3^) on 2″ *c*-plane sapphire substrates.^27–29^ All experimental details are described in Figure S1. To this end, single crystal wurtzite structure NRs were obtained with a growth direction on the [0001] c-plane (Figures S2 and S9).^29^ GaN NRs with different diameters (such as 900 nm, 800 nm, 500 nm and 200 nm) were obtained under the same etching conditions (except etching time), and thus, no diameter dependence of the unintentional defects’ incorporation was expected (see SI for more info, Figure S5). These GaN NRs are intentionally n-type doped (silicon doping), with a majority carrier (electron, n̅ ) density of ∼10^17^ cm^–3^, determined from our previous electrical measurements.^30–33^ The electron density is comparable to those reported in high-quality MOVPE-grown GaN layers.^30–33^

For SPV and SPS measurements, the GaN NRs on sapphire were fixed on grounded holder with copper pins contacting the top layer of GaN, as shown in Figure S11. Measurements were performed using a KP (with stainless steel probes, see SI) under dark and illuminated conditions with seven monochromatic diodes and white light inside a sealed nitrogen cell (Figure S2).

Symmetric dark and light steps, with a millisecond resolution with different wavelengths (300 nm–1000 nm), were implemented to realize a momentary nonequilibrium state under a low-level optical illumination in sub- and super-bandgaps and to analyze the carrier density distribution at the surface as a function of time. To this end, the corresponding change in the surface potential resulted from some charge transfers and redistributions within the defects (bandgap states).^34^ Note: the sub-bandgap illumination (i.e., hυ < E_g_) illustrates a charge transfer between defects and bands (Figure 1c), while super-bandgap illumination (i.e., hυ > E_g_) illustrates a charge transfer from band-to-band (Figure 1b), i.e., from the valence band (E_v_) to the conduction band (E_c_).^34^ SPS is an effective method that analyzes surface states’ energy position and nature (Figure 2) and demonstrates the contact potential difference (CPD) values of GaN NRs in the dark and under optical illumination (sub- and super-bandgap illumination). The photoinduced charge carrier excitation between surface states and bands introduces distinct features in the SPV spectrum at the energy threshold of individual transitions. Electron excitation from the surface state to the E_c_ generates +SPV (−ΔCPD), while hole excitation to the E_v_ results in −SPV (+ΔCPD). On scanning from dark to light (*Y*-axis), the CPD in the light shows a negative quasi-Fermi level (−ΔCPD) shift with respect to the dark, which equals + SPV, i.e. when scanning from super-bandgap energy of ∼4.5 eV to 3.34 eV. Therefore

1 −ΔCPD=+SPV

This is characteristic of an n-type semiconductor with acceptor-like defects and demonstrates the dominant carrier excitation mechanism.^34^ Therefore, the defects, which are in the m-plane, as will be shown later, are acceptor-like surface states (Q_ss_) and become neutralized by hole diffusion (minority carrier, p̅) to the surface, together with the excitation of electrons from Q_ss_ to the E_C_, i.e., a depopulation mechanism (Figure 1c).

**Figure 2:**
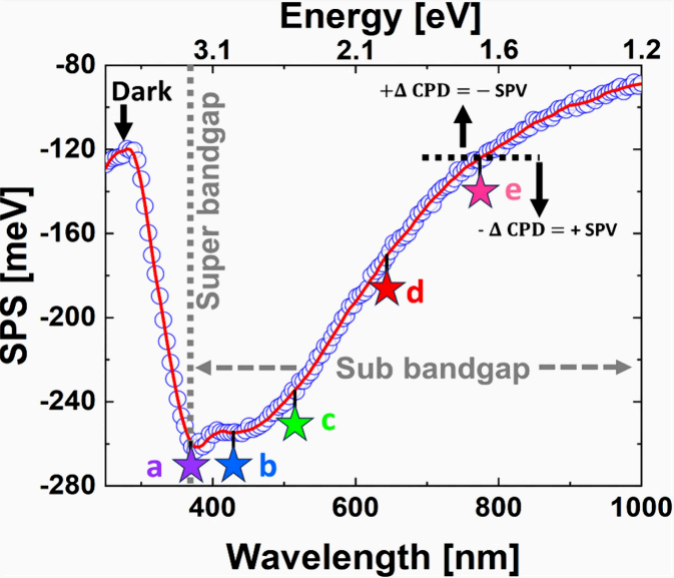
SPS spectra of GaN NRs scanning from dark to super- and sub-bandgap energies. (a) Band gap (E_g_) = 371 nm (3.34 eV). (b) Saturation of photovoltage indicating the acceptor states being filled (population from 411 to 455 nm). (c, d, e) Acceptor surface states within the excitation wavelength of 545, 700, and 850 nm (e), considering dark CPD as the reference point above which the photovoltage inversion occurs (+ΔCPD = −SPV).

Upon scanning from super- to sub-bandgap energies (i.e., from ∼4.1 to 1.2 eV, see top *X*-axis), revealed a distinct SPS signal as a sharp knee around ∼371 nm (∼3.34 ± 0.10 eV), which represents the bandgap of the GaN NRs (Figure 2). At super-bandgap illumination, we have a high absorption coefficient near the bandgap (E_g_).^35,36^ However, the SPS signal starts to decrease when we shift to higher wavelengths (from 270 to 390 nm). This is attributed to (i) the absorption of low energy photons slightly below E_g_ resulting from the Franz-Keldysh effect,^36^ and (ii) photoexcitation of charge carriers from defect states (shallow states) to a band, or vice versa, which in turn, may cause an error in precisely defining the bandgap energy (±100 meV). The negative CPD change from 283 to 371 nm in the SPS spectra clearly shows band-to-band transition. A minor positive CPD slope change followed by photovoltage saturation from 411 to 455 nm is due to the photoinduced population of acceptor surface states situated (∼0.64 eV above the E_v_).^34^ Above point (e) in Figure 2, an inversion in ΔCPD (-SPV) corresponds to an increase in the surface barrier height and is believed to be due to a photostimulated population of the surface states.^37^

Unlike bulk GaN, which shows a constant Q_ss_ along the sub-bandgap emission (Figure S3, Supporting Information), the Q_ss_ in GaN NRs, shows exponential decreases from near the band-edges (shallow states) toward the midbandgap (deep defects), with notable ΔCPD ≈ 141 meV, from −120 meV to −261 meV (Figure 2, see *Y*-axis) relative to ∼5 meV for bulk GaN (Figure S3). These defects in GaN NRs may arise from the etching process (ICP-RIE) and can either boost charge transport (surface doping) or impede it by trapping or scattering the carriers; they may also be radiative or nonradiative defects. To probe these defects’ properties, we monochromatically illuminated the sample within a range of the sub-bandgap energies, i.e., from 400 to 850 nm in five different wavelengths (from a to e) and referenced with two wavelengths of light in near IR (which gives SPV ≈ 0, f and g), as shown in Figure 3a.

**Figure 3:**
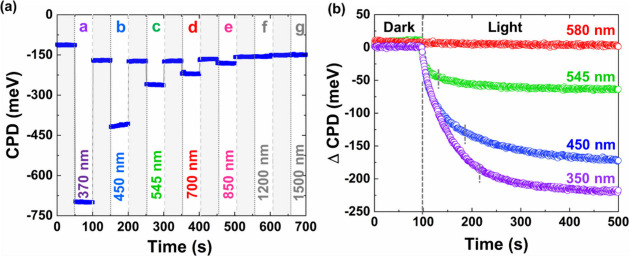
(a) SPV (−ΔCPD) of the GaN NRs at low optical monochromatic illumination. (b) ΔCPD after reaching equilibrium with the corresponding wavelength of light.

Figure 3a shows the SPV signal of the GaN NRs (ΔCPD in the dark and light under low optical monochromatic illumination), i.e., weak injection, as a function of time. Given the GaN’s carrier lifetime on the order of tens of nanoseconds, the low optical power density (∼0.3 Wcm^–2^) used in this study leads to a photogenerated free carrier concentration of electrons (n̂) and holes (p̂) of about ∼10^13^–10^14^ cm^–3^. In this case, the photogenerated carriers are equal, i.e., n̂ = p̂. This is significantly lower than the equilibrium electron density (∼10^17^ cm^–3^), which confirms the weak injection condition, i.e., p̅ ≪ p̂ = n̂ < n̅.^38,39^ Regardless of the irradiated wavelength, at time, t = 0, i.e., after immediate light exposure, we obtained + SPV, which again confirms the n-type characteristic of the GaN NRs. However, by considering the semiconductor weak injection theory, we note that the response time of the GaN, τ of the minority carriers in our case, τ_*h*_, was surprisingly not constant (∂p̂/∂t ≠ 0) and was found to be related to the irradiated wavelength (Figure 3b).^38,39^ The weak injection approach has been used to characterize photoconductive detector quality in slow and fast pulses.^38,40,41^ At a longer wavelength (points c-e), we observed τ_*h*_ < t, which can explain the low obtained SPV signal, which should be proportional to G_opt_·τ_*h*_; and ∂p̂/∂t = 0 at the scale of ∼ millisecond (Figure 3b). The SPV theory also supports these results in which the SPV becomes saturated at the millisecond scale and is proportional to 1/λ_ex_. However, in the blue regime (short wavelength, high frequency, points a and b), we noticed two unexpected phenomena: (i) the lifetime of the excess photogenerated minority carrier is greater than the irradiation time, i.e., τ_*h*_ > t, which explains the high obtained SPV signal, on one hand (Figure 3b). However, on the other hand, we noticed that ∂p̂/∂t ≠ 0, even after several tens of seconds (marked as a dashed vertical line in Figure 3b).

Surprisingly enough, we observed, in the blue regime, (ii) a positive gradient of the CPD slope change (Figure 4a), illustrating a momentary reverse mechanism (population mechanism, p-type character) due to the photostimulated population of the surface states, which was previously explained for the SPS spectra. To our knowledge, this is the first time we have seen this effect on the surface. The slope of the p-type represents the impact of the generated electrostatic field as will be discussed in next section.

**Figure 4:**
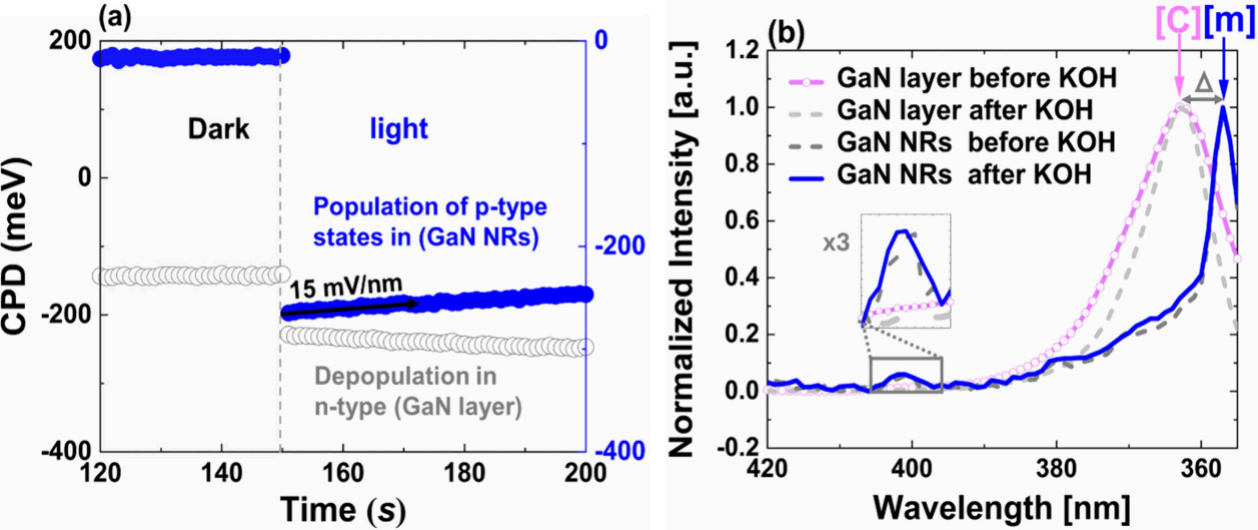
(a) A decrease in positive SPV in blue light for GaN NRs with time, indicative of a population of acceptor defect states; a pristine n-type GaN layer showed no such effect. (b) A PL emission peak from GaN NRs in the blue regime is shown. [c],[m] denotes the crystal planes (see S2). Note: The GaN NRs were removed from the intact places and were flattened on the surface to measure the electronic properties. More information can be found in the SI and Figure S9.

Considering these observations, the p-type surface state can be explained by factors including strain-induced defects.^42–48^ In our case, the p-type surface states observed within the energy, E_s_, of ∼1.54 eV are above the E_v_. When the empty p-type state (acceptor) was excited with photon energy in this range (above point (e) in Figure 2), the photogenerated electrons tended to accumulate at the surface rather than being swept away by the space charge region (SCR) electric field (Figure 1c). Therefore, the SPV signal started to develop as there was a significant diffusion of the photogenerated electrons, i.e., onto the surface against the built-in-electric field (i.e., SCR). Therefore, band bending (BB) developed, contrary to the usual cases, which illustrates a photovoltaic inversion (Figure 2 and Figure 4a). This phenomenon occurs mainly in empty p-type defect states located close to the valence band and can only occur if the surface BB is small and in thermal equilibrium. In other words, the strain-induced defects generate an internal electrostatic field that is opposite to the SCR field and generates the additional photovoltage (but in the opposite direction) that overcomes the BB. As a result, there is a minor net electric force, which can redistribute the carriers. This occurred in the sub-bandgap energies; therefore, we have an intercharge transfer between the defect and the E_v_. This can be explained by quasi-neutrality or/and a quasi-Dember effect at the surface by the following two steps: (i) at t = 0, immediately after light exposure, the surface state captures the photogenerated holes, p̂, and becomes neutral and reduces the BB. However, (ii) at *t* > 0, after a quasi-equilibrium, the thermalized holes, p̅, drift by the electrical field generated by the defect. The field starts to build up potential against the n-type nature of the bulk material and forms a p-type defect character (Figure 4a). A similar effect has been reported in bulk GaAs with minimal BB energy at the surface.^41^

To prove that the strain-induced this defect, we first compared the SPV under blue light exposure, and then we calculated the additional SPV signal from the generated electrostatic field (Figure 4a). For the first step, the bulk layer of the GaN showed n-type character and ∂p̂/∂t ∼ 0 (Figure 4a). We used the most updated density functional theory (DFT) calculations for the second step and compared them to our experimental results.^49^ In general, all compounds of the wurtzite crystal structure, such as GaN exhibit a spontaneous polarization (bulk dipole density) P_0_ under an external electric field (or defect) and points opposite to the crystal structure’s axis. Following the common sign conventions, we will denote the component P_0_ along the c̅ axis by P, i.e., in the usual case, *P* < 0. Due to the texture of the GaN NRs, an average dipole density is P_*z*_ = αP, while P_*x*_, P_*y*_ = 0 for symmetry reasons. Following simple electrostatic concepts, an electric field is equal to

2 $$F_{z}={\left(\varepsilon \varepsilon_{0}\right)}^{-1}P_{z}$$

where ε is the static dielectric constant of the GaN, and ε_0_ the vacuum permittivity.^49^ In addition, due to the weak injection, we have a quasi-neutralization case, and therefore, the internal field is also zero (no BB). This supports our conclusion that the gradient in the SPV, i.e., the p-type character in the blue regime, occurs only due to the electrostatic field effect (Figure 4a).

We were able to resolve this effect for the first time, according to the authors’ knowledge, in GaN NRs in the blue regime by the population mechanism upon external perturbation, mainly through the strain-generated electrostatic field (see Raman measurements in the SI, Figure S10). The spontaneous polarization of wurtzite material is usually inferred by calculating the electrical field discontinuity at the interfaces in a periodic stack of hexagonal wurtzite (h) and cubic zincblende (c) type layers of the respective compound by DFT. Since P_0_ = 0 in the cubic zincblende layers, for symmetrical reasons, P_0_ of the wurtzite layers in the GaN NRs can be directly extracted from that field discontinuity, i.e.

3 $$\Delta F=F_{h}-F_{c},\mathrm{when}\;P_{0}=-\varepsilon \varepsilon_{0}\Delta F$$

We adopted the most recent DFT calculation of Romanov, Baker, Nakamura, and Winzer et al. for the bulk GaN. We extracted F_T_ = 2.14 × 10^8^ Vm^–1^ = 214 mV nm^–1^ and P_T_ = −0.017 Cm^–2^ using *ε* = 8.9 for the compounds of field and polarization parallel to the crystal c̅ axis.^50^ We use the subscript (T) to explicitly mark this as the theoretical value for a single crystal GaN. This value is about one factor lower than typical polarizations of GaN wurtzite semiconductors (P_T_ = −0.029 Cm^–2^). However, the P_T_ is much more pronounced in GaN NRs by a factor of 3 and in the opposite direction P_T_= +0.051 Cm^–2^, leading to a reversed population (i.e., depopulation) in the SPV signal.

Moreover, the photoelectrons in blue light (and super-bandgap illumination), associated with the SPV, are excited in the space charge region (SCR); thus, the photocarrier (p̂ = n̂) senses the electronic field due to the polarization. Therefore, a corresponding blue shift in the GaN NRs threshold relative to GaN layer is also observed (Figure 4b). From the slope of the data plot (Figure 4a), we can extract a value of F_*z*_ = +15 mV/nm for this field in GaN NRs. A comparison with the calculation yields F_*z*_ = 0.07 F_T_ (when F_T_ = 214 mV nm^–1^). We can translate this to the gradient in SPV (due to the electrostatic effect), which also shows a 7% gradient relative to the SPV signal (Figure 4a). The weak electrostatic field leads to poor electron–hole overlap, which leads to low PL (compared to band-to-band, Figure 4b).^3^ This effect cannot be observed in the deep defects as the polarized electrical field fades by 1/r^3^ or in GaN layers. Therefore, the strain-induced electrostatic field generates shallow defects and is solely observed in the blue regime and at the surface. Finally, the generated electrostatic field is supported by observing a blue shift, Δ, in GaN NRs relative to the GaN layer, from 3.39 ± 0.01 eV to 3.46 ± 0.01 eV (theoretical E_g_ = 3.4 eV as in bulk GaN), regardless of the KOH passivation (Figure 4b). This shift was not observed in the bulk GaN (Figure 4b). This Δ confirms that the PL transition energy GaN layer (at 3.39 eV) corresponds to the c-plane, whereas the PL for GaN NR (at 3.46 eV) corresponds to the m-plane. The induced strain shows that the c-plane emits 10 meV below and the m-plane 60 meV above the GaN bandgap, respectively.^3^ Considering the above-mentioned, it is interesting to note that this effect occurs at room temperature, i.e., the electrostatic field with thermal generation overcomes the built-in barrier, as illustrated in (Figure 1c). An applicative case of the strain induces a p-type defect when optical transitions occur between a defect level and bands, generating luminescence.^51^ To precisely follow the p-type defect’s luminescence characteristics, we measured the PL in the blue regime and band-to-band transitions combined with KOH passivation (see experimental section in SI for more details). Under the weak injection parameters, the exciton (i.e., electron–hole couple attracted by the Coulomb force) and carrier transitions between surface states and bands are surface-related within the absorption coefficient regime (i.e., d ≈ a^–1^). Therefore, electron transfer from SCR to the defects in the blue regime is mainly due to the electrostatic field.

As illustrated in Figure 4b, the GaN NRs show a stable blue emission (at RT and under ambient conditions) in the blue regime, regardless of the KOH passivation. In contrast, the bulk GaN shows no blue emission either before or after KOH passivation. Since the hydroxylating reaction occurs due to the oxidation of the Ga top atoms to Ga–O by OH^–^ ions, instead of N atoms,^52^ we can claim that the strain effect generates shallow defects solely from Ga defects occupied by Si (SiGa) or Ga–O (more details of possible scenarios for nature of the defects are shown in the SI and Figure S6).^5^ In addition, the combination of KOH passivation and PL helped us discriminate between strain-induced blue emission and contamination/chemical vacancy-induced PL with the possible transition occurring between the contaminated C_Ga_ (carbon gallium sites) as a donor and C_N_ (carbon–nitrogen sites) as an acceptor.^53^ This was disproved in our case by the dramatic decrease in the C 1s signal (representing contamination) by 50%, from 1 to 0.5, after KOH hydroxylation passivation (Figure S4, Supporting Information). All relevant XPS details on N 1s, O 1s, Ga 2p, and Ga 3d are found in SI, Figures S7 and S8. After KOH passivation, we observed a slight narrowing (∼5 nm) in the tail and full width at half-maximum (fwhm) near the band edge PL peak of the GaN bulk and NRs. fwhm occurred due to multiple radiative transitions of minor shallow defect states near the edge emission (i.e., band-to-band transition). The slight sharpening is due to the passivation of defects originating from Ga vacancies (shallow defects, Figure 4b).

In summary, strain generates an electrostatic field that momentarily inverts the carrier mechanism (from depopulation to population) and realizes p-type defects in the m-plane, which are responsible for blue light emission in GaN NRs. Utilizing p-type defects on n-type GaN to create p–n junctions is a potentially valuable concept for various applications and devices. Although this structure is still in the proof-of-concept stage, its excellent performance, stability, reproducibility, and potential scalability suggest promising prospects in high-power and high-frequency electronics, solid-state lighting (such as light-emitting diodes, optical switches, and portable electronics), molecular electronics, smart hybrid sensors, and in other fields and devices.

## Supplementary Material



## Notes

GaN etching, characterization of GaN, surface photovoltage, Kelvin probe method, Raman, PL, XPS, and KOH termination (PDF)
