# Supplementary material for: p‑Type Surface Defects on n‑GaN Nanorods
Source: Nano Lett. 2025 May 19;25(22):9118–24. doi: 10.1021/acs.nanolett.5c01839 (PMC12142676; doi:10.1021/acs.nanolett.5c01839)
Supplement: Supplementary file 1 [file nl5c01839_si_001.pdf]

Supplementary Information

p-type Surface Defects on n-GaN Nanorods

Sumesh Sadhujan, [1,†] *Sherina Harilal,*[1,†] *Kefan Zhang,*[1] *Riam Abu Much,*[2] *Abdullah AbuBekr,*[1] *Ayat Asleh,*[1] *Awad Shalabny,*[1] *Amro Sweedan,*[3] *Nursidik Yulianto,*[4,5] *Andam Deatama Refino,*[4,6] *Hutomo Suryo Wasisto,*[4,7] *Laila Abu Madegam,*[8] *Aeid Igbaria,*[8] *Mariela J. Pavan,*[3] *Muhammad Y. Bashouti* [1,3*]

[1]*Department of Solar Energy and Environmental Physics, Swiss Institute for Dryland Environmental and Energy Research, J. Blaustein Institutes for Desert Research, Ben-Gurion University of the Negev, Midreshset Ben-Gurion, Building 26, 8499000, Israel.*

[2]*The Academic Arab College for Education in Israel-Haifa, 22 Hahashmal St. Haifa 33145, Israel.*

[3]*The Ilse-Katz Institute for Nanoscale Science & Technology, Ben-Gurion University of the Negev, POB 653, Marcus Family Campus, Building 51, Beer-Sheva 8410501, Israel.*

[4]*Institute of Semiconductor Technology (IHT) and Laboratory for Emerging Nanometrology (LENA), Technische Universität Braunschweig, Braunschweig 38106, Germany.*

[5]*Research Center for Photonics, National Research and Innovation Agency (BRIN), Kawasan Puspiptek Gd. 442, South Tangerang 15314, Indonesia.*

[6]*Engineering Physics Program, Institut Teknologi Sumatera (ITERA), Jl. Terusan Ryacudu, Way Huwi, Lampung Selatan, Lampung 35365, Indonesia.*

[7]*PT Nanosense Instrument Indonesia, Umbulharjo, Yogyakarta 55167, Indonesia.*

[8]*The Department of Life Sciences, Ben Gurion University, Beersheba, 841050, Israel.*

[†]*Contributed equally.*

[*]*e-mail: Bashouti@bgu.ac.il*

Fabrication of n-GaN nanorods

The epitaxial GaN films with a thickness of ~4.8 µm were grown by MOVPE on 430 µm thick 2″ *c*-plane sapphire substrates (Fig. S1a). Initially, a 2.45 µm thick Si-doped GaN layer with a doping concentration of 10^{17} cm^{-3} was grown, followed by a 1.34 µm thick unintentionally doped GaN film. Finally, a 1 µm thick *n*-GaN layer was grown with a doping concentration of 10^{17} cm^{-3}. The *n*-GaN was realized using monosilane (SiH_4) as a Si precursor in an Aixtron AIX2600HT G3 24 × 2” planetary reactor. The GaN nanorods were fabricated by a top-down fabrication method combining UV-photolithography using an MJB4 mask aligner (SÜSS MicroTec SE, Germany) and inductively coupled plasma reactive-ion etching (ICP-RIE). After creating circular photoresist masks on the top of GaN surfaces, electron beam evaporation was performed, followed by a chemical lift-off process to deposit the 300 nm thick Cr layer serving as an etch mask for creating GaN nanorods (see Figs. S1b and S1e). Subsequently, a wet chemical etching was employed (see Ref.16–19, for more experimental details). In the hybrid

etching sequence, the first physical etching of GaN films was carried out using an SI 500 C plasma dry etcher (SENTECH Instruments GmbH, Germany) to define the height of vertical GaN NRs (Fig. S1c). Afterward, we treated the sidewalls and trapezoidal shape using a wet chemical etching process using AZ400K at 90 °C for 6 hours to obtain a smaller diameter (Fig. S1d). Again, wet etch duration was controlled to shrink the rod diameter down to 500 nm (see Figs. S1f and S1g). As a final step, Cr masks were removed entirely using Etch18 Cr etchant for 5 min prior to surface spectroscopy investigation.

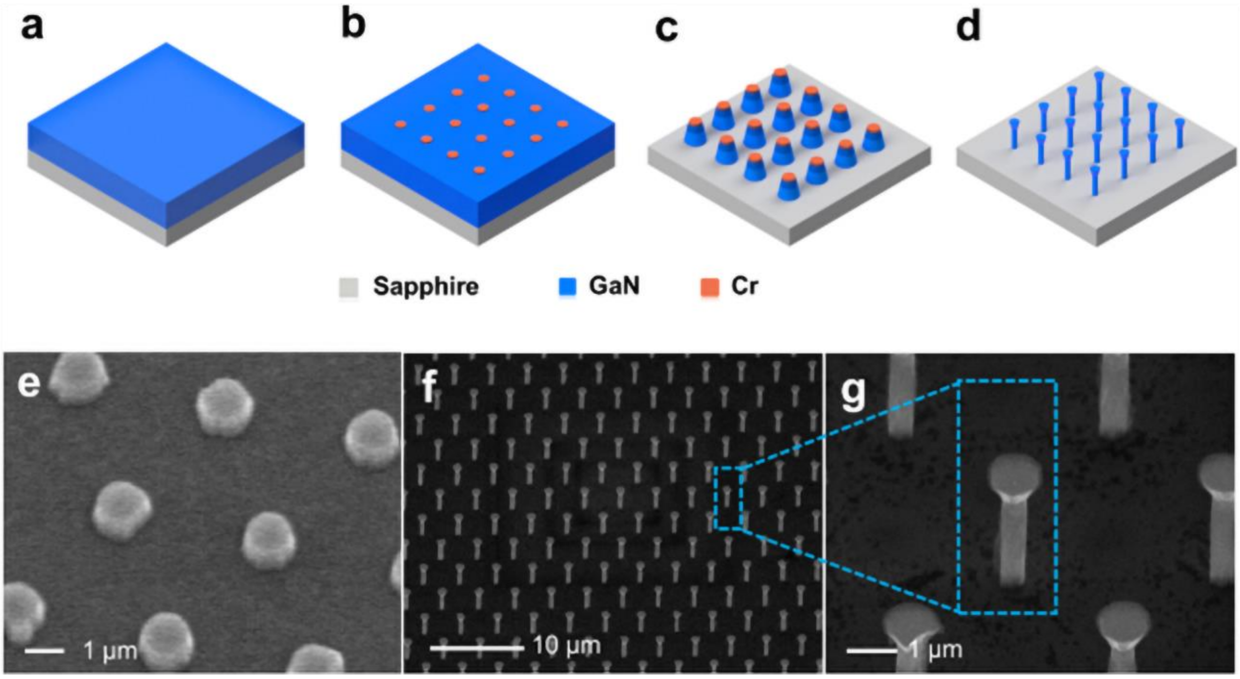

Figure S1. Top-down fabrication of vertical GaN nanorod arrays. (a) Growth of bulk GaN by MOVPE. (b) Photolithography to pattern chromium (Cr) masks on GaN surface. (c) ICP-RIE of masked GaN resulting in trapezoidal GaN nanorods with rough sidewall surface(d) KOH-based wet chemical etching of GaN nanorods as a post-processing treatment after ICP-RIE to shrink the nanorod diameter and smoothen the sidewall. (e) Patterned Cr masks on planar GaN. (f, g) Vertically aligned GaN nanorod after hybrid etching processes (i.e., ICP-RIE and KOH etching).

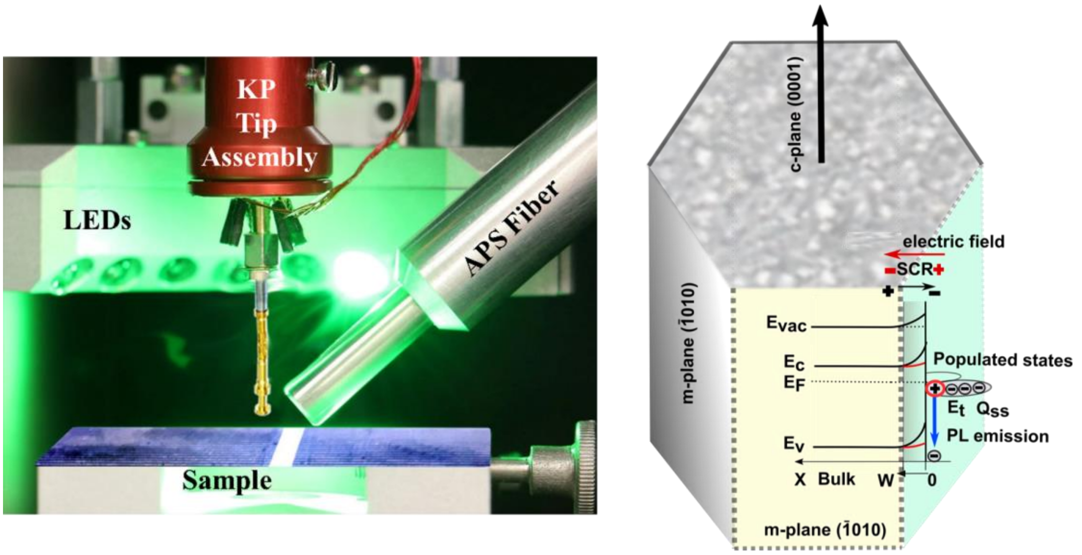

Figure S2. (a) Kelvin probe test assembly for surface photovoltage spectroscopy, (b) Schematics of band diagram showing radiative emission of electrons from populated surface defect states.

Electrical measurements conducted on n-GaN nanorods

A Kelvin probe system (APS04, KP technology) with a stainless-steel tip diameter of 2 mm performs surface photovoltage (SPV) and surface photovoltage spectroscopy (SPS) measurements. The tip work function (4.64 eV) was pre-calibrated with a freshly cleaved, highly ordered pyrolytic graphite (HOPG). A quartz tungsten halogen (QTH) has a variable intensity light source (Fiber lite DC-950) coupled with a spectrometer that provides wavelengths ranging from 200 nm to 1000 nm for measuring SPS. Uniform illumination on the sample with the same light intensity was ensured, along with a large illumination spot size of ~10 mm compared to the tip diameter (2 mm). For all Kelvin probe measurements, the tip distance was also kept constant.

The silicon-doped GaN nanorod arrays were illuminated with wavelengths ranging from 250 nm to 1000 nm (step size 5 nm, delay time of 1000 ms). The samples with insulating sapphire substrate were grounded using a grounded holder with copper pins contacting the top layer of GaN, as shown in Fig. S10. During the SPS measurement, a positive surface photovoltage

(SPV) on the semiconductor surface, such as -ΔVc = SPV, indicates an n-type semiconductor characteristic.

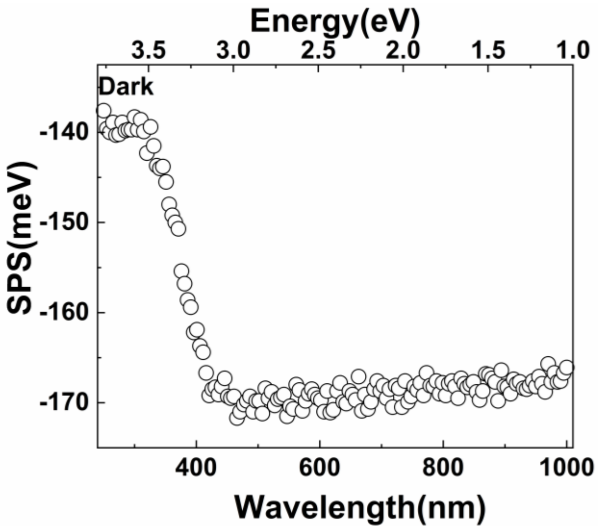

Figure S3. SPS spectra of bulk GaN, scanning from dark to illumination under super and sub-bandgap energies.

XPS measurements

XPS was performed by irradiating and exciting the sample core level by monochromatic Al Kα (1487 eV). The photoelectrons were collected at an angle of 55º from the surface for GaN bulk film and GaN NRs arrays, enhancing the surface sensitivity. The survey spectra were scanned from 0-1360 eV, and emission peaks were collected with a pass energy of 20 eV and a step size of 0.1 eV. The peaks were deconvoluted using the software package XPSPEAK version 4.1. Peak fittings were obtained for $\chi^2<1$, where χ^2 is the standard deviation.

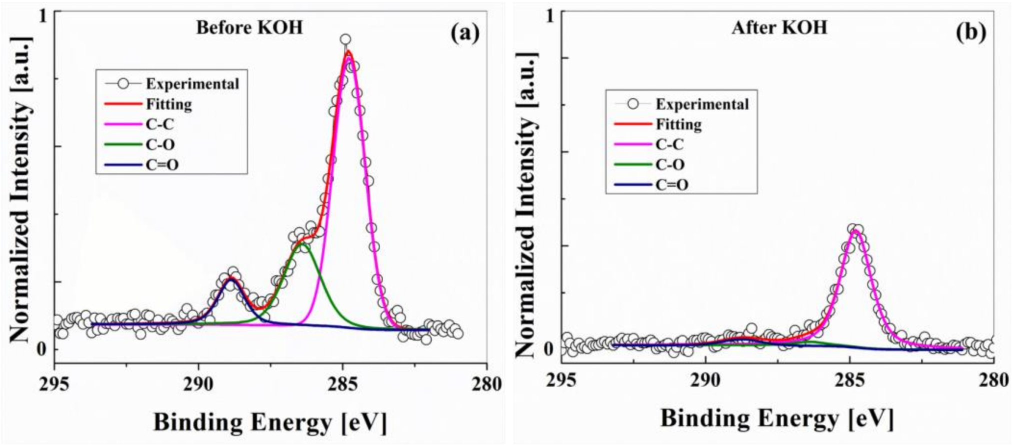

Figure S4. Deconvoluted XPS peaks C1s peak of (a) GaN NRs (b) KOH etched GaN nanorods.

Effect of KOH etching on the diameter of GaN rods and SPS

The different diameters used in this research are 900 nm, 800 nm, 500 nm, and 200 nm (as mentioned in main manuscript at page 5). All the different diameters show p-type characters at the same wavelength (450nm). The different diameters show the same tendency of the SPS and SPV. As an example, we show in Fig S5 the SPS of the thinnest GaN NRs (225nm) after KOH etching (5M, 30 sec). The Figure shows the defect distribution near the bandgap which shows p-type density with similar tendency as the 500nm Ga NR, i.e., here we also observe the same mechanism of photovoltage inversion as observed Ga NR (500 nm), but the density of the p-type is not the same. The figure shows that the ΔCPD (-SPV) seems to decrease, indicating less p-type defect density spread near the band gap.

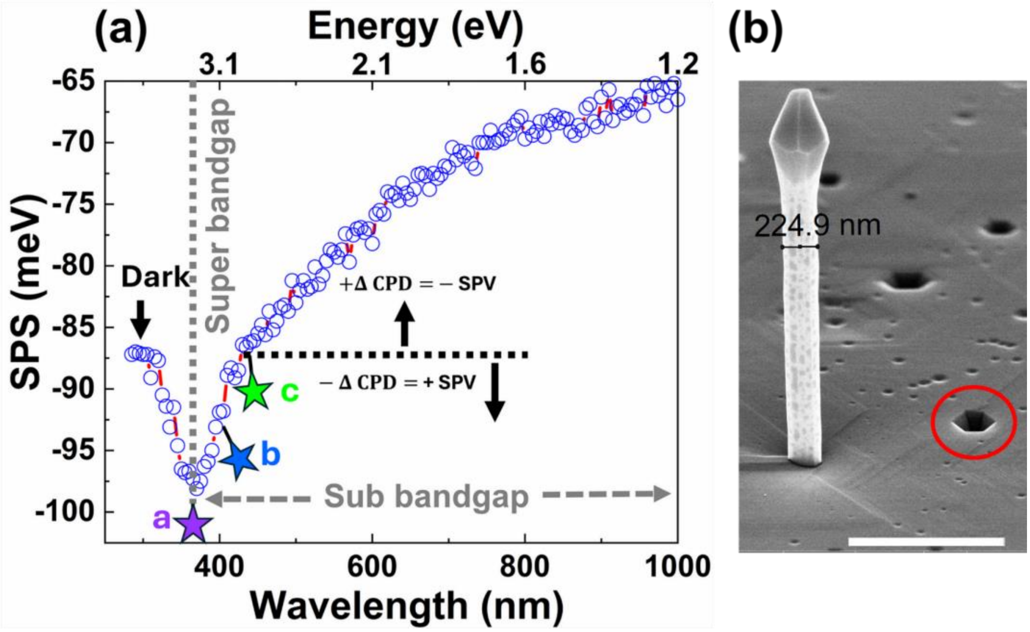

Figure S5. SPS spectra of a GaN NRs (~225 nm diameter after KOH etching) scanning from dark to super and sub-bandgap energies (a) Band gap (E_g) = 365 nm (3.39 eV) (b) depopulation of acceptor surface states at an excitation wavelength of 410 nm with + SPV (c) considering dark CPD as a reference point the photovoltage inversion occurs above it (+ΔCPD = -SPV), (b) SEM image of a Ga NRs (KOH etched shrink diameter to ~ 225 nm). Scale bar 1 μm.

Nature of the defects

The Si-doped n-type GaN has been KOH etched, and the nature of these defects (p-type) are (i) gallium vacancy (V_{Ga}), (ii) occupied vacancy by Si, (Si_{Ga}), (iii) occupied vacancy by residual impurities or incorporated species such as oxygen, (V_{Ga}-O), and (iv) carbon substation on the nitrogen site (C_N).

For (i), i.e., point defects, e.g., gallium vacancy, the (V_{Ga}) is formed in *n*-type GaN by etching (wet KOH etch, 5M for 30 sec, or dry etch with reactive ions) that can preferentially remove Ga atoms from the surface and increase the concentration of Ga vacancies. Since a missing Ga atom leaves behind an electron deficiency, V_{Ga}acts as a deep-level acceptor state. For (ii), Si can occupy the V_{Ga} in the gallium vacancy, which becomes Si_{Ga} and is considered a shallow donor or acceptor (amphoteric nature). For (iii), the Ga vacancies can interact with residual

impurities or incorporated species. For instance, complexes like V_{Ga}-O (where oxygen from the etching solution or ambient is involved) or even complexes with Si may form. For (iv), carbon can be substituted on the nitrogen site C_N and exhibit acceptor-like behavior. Each defect introduces states near or within the bandgap that can capture electrons, thereby mimicking or contributing to p-type behavior even in n-type material.

From all these reasons, we conclude that the p-type (acceptor defect) generated is predominantly due to the first reason, i.e., point defects generated by gallium vacancy (V_{Ga}) by formed from KOH etching. The states formed due to Si_{Ga} are not significant compared to V_{Ga}, moreover, the defects state due to carbon (C_N)and oxygen (V_{Ga}-O) are not reasonable since they were minimized by 50% in the etched GaN NRs (confirmed from XPS analysis), while the p-type value remains the same value and energy position.

Photoluminescent (PL)

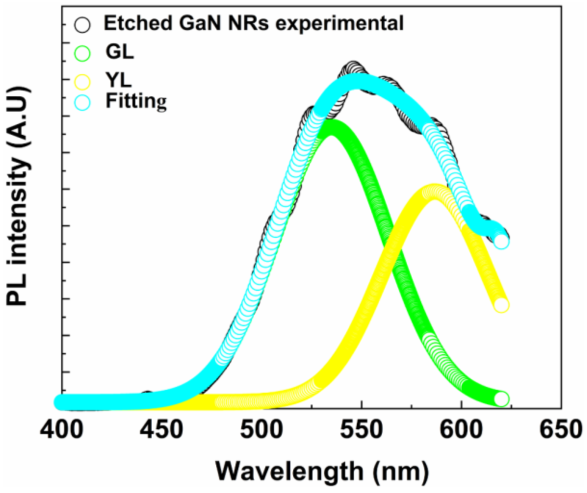

Figure S6. Deconvoluted PL spectra of etched GaN NRs showing emission peaks from defect Green (GL), and Yellow (YL)

Photoluminescent (PL) due to transitions between shallow donors to acceptor-like deep levels is shown in Fig. S6. The PL spectra show a broad yellow luminescent peak (YL) with maximum

intensity at ~580 nm (~2.1 eV). In addition, green luminescence (GL) was also observed and could arise from transitions between deep acceptor levels to the conduction band. These luminescence properties of defect transitions were reported by H. C. Yang *et al.* and by Michael A. Reshchikov *et al.*[1,2] A double structure commonly assigned to GL with peak splits can vary in intensity in doped samples, leading to broadband with shoulder and overlaps with the YL at room temperature, as observed clearly in our GaN NRs arrays, Fig. S6.

XPS elemental and Valence band emission analysis of GaN NRs with surface defects.

Fig. S7 a,b shows the evolution of the valence band for n-GaN before and after etching into GaN NRs. The results indicate that the valence band maximum (VBM) of the bulk n-GaN is located 2.46 eV below the fermi level (E_F). A significant shift in VBM towards a lower BE of 1.72 eV was observed for KOH-etched GaN NRs.

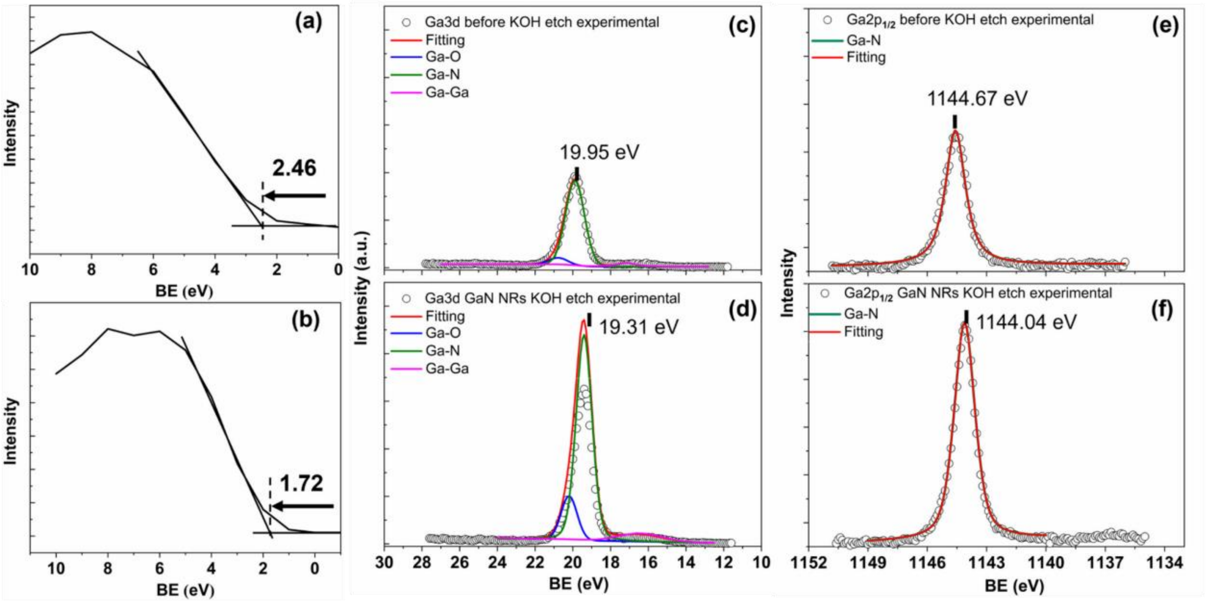

Figure S7. The XPS spectra of the valence band for the (a) bulk n-GaN (b) KOH etched GaN NRs (c) Ga3d states for bulk n-GaN, (d) Ga3d states KOH etched GaN NRs (e) $Ga2p_{1/2}$ states bulk n-GaN (f) $Ga2p_{1/2}$KOH etched GaN NRs

Fig. S7 c,d shows the position of the Ga3d peaks, which are deconvoluted into three peaks: metallic gallium, gallium nitride, and gallium oxide. On analyzing the Ga3d peak, a shift

similar in trend to the VBMs was observed, from a high BE of 19.95 eV (for bulk n-GaN) to 19.31 for the etched GaN NRs **(**Fig. S7d**)**. The energy difference between Ga3d and VBM was ~ 17.5 eV before and after etching, indicating the BE shifts characteristics of trapping (electron or hole) by defect states, creating localized electrostatic charges on the surfaces. A similar trend in shifts towards lower BE is also observed for Ga2p peaks for the etched GaN NRs compared to bulk n-GaN, as shown in Fig. S7 e,f. From Fig. S7, one can observe a steep onset in VBM for etched GaN NRs in addition to the shift in VBM, which could result from the reduction in the carbon and oxygen contaminants on the surface, as reported by D. Majchrzak *et al.* [3]

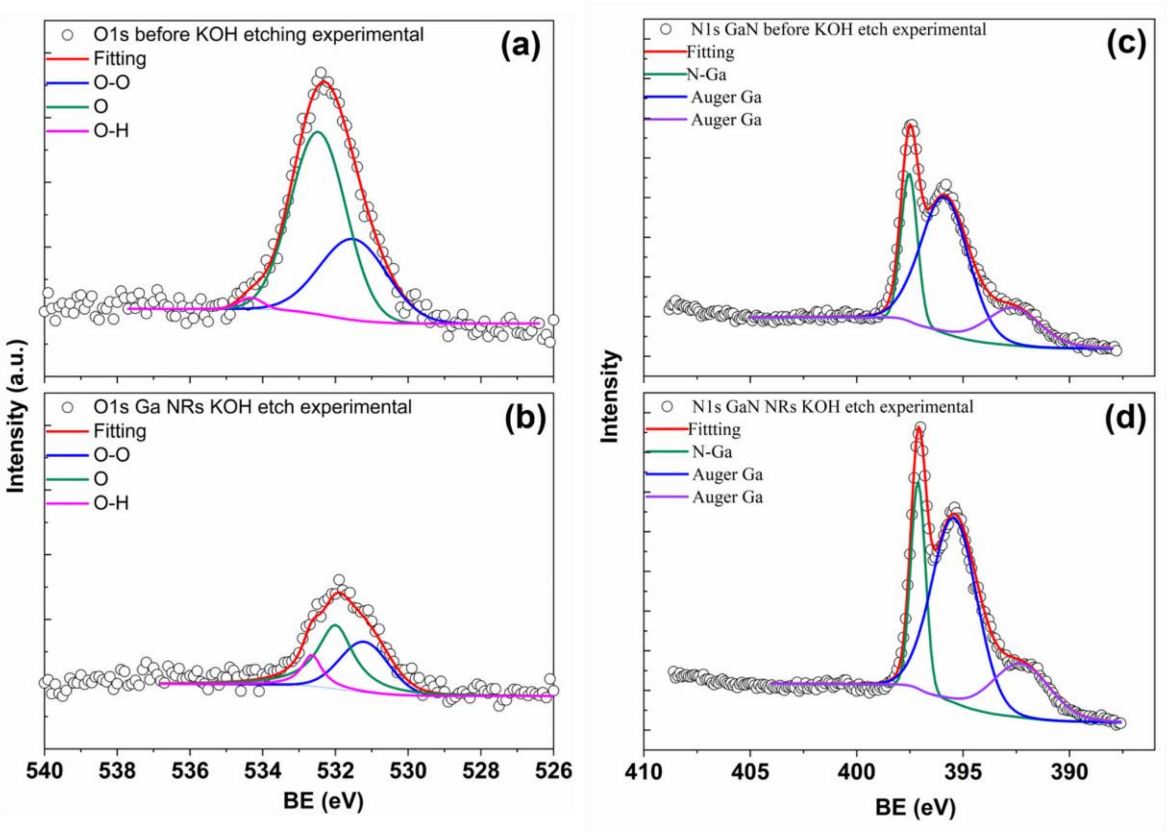

Figure S8. The XPS spectra of the (a) O1s bulk n-GaN (b) O1s KOH etched GaN NRs, (c) N1s states for bulk n-GaN and (d) N1s KOH etched GaN NRs

The O1s spectrum is deconvoluted into three peaks attributed to metal oxide, chemisorbed oxygen atoms (originating from contamination in the air), and hydroxide, respectively, as in Fig. S8 a,b. A significant decrease in O1s (metal oxide, chemisorbed oxygen atoms) peak area is noticeable on KOH-etched GaN NRs; however, the hydroxide peak increased compared to

bulk n-GaN. This could be the consequence of the OH^{-}ions attacking the back bond of Ga atoms (tetrahedrally coordinated), oxidizing the gallium atop atoms on the m and a-planes in GaN NRs to gallium oxide, which is soluble in an aqueous KOH alkali solution, making GaN NRs thinner in diameter. A similar OH^{-}mediated etching of GaN polar surfaces was investigated by Dongsheng Li. *et.al.*[4] The N 1s spectrum is deconvoluted into three peaks attributed to N-Ga and two auger Ga peaks as in Fig. S8 c,d.

GaN after KOH (5M, 30 sec)

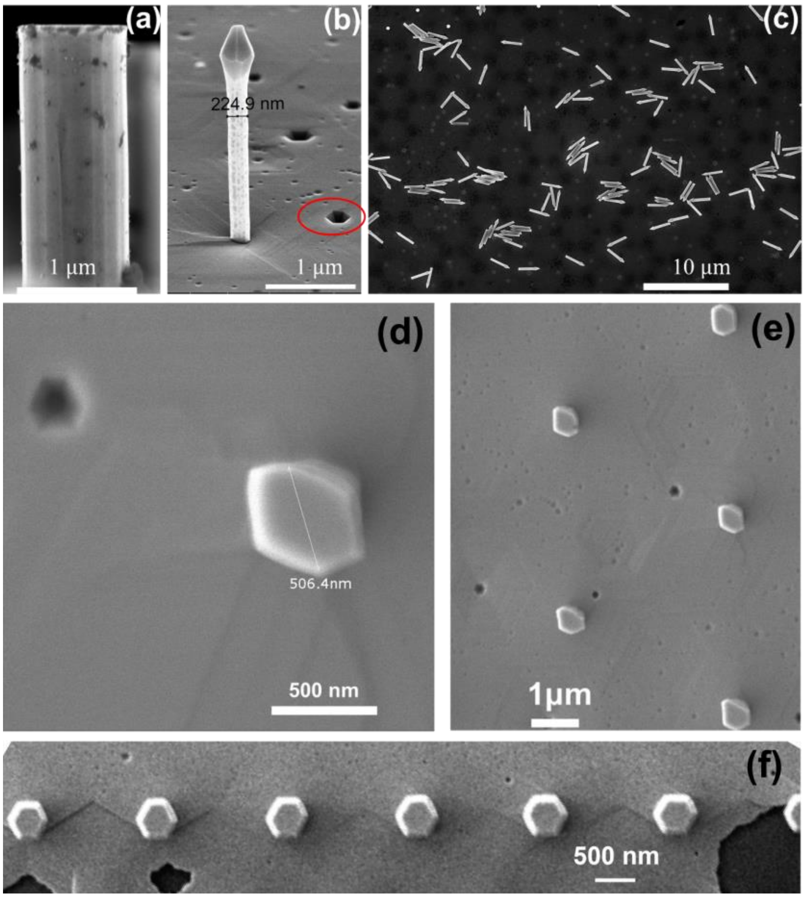

Figure S9. Cross-section SEM image of (a) GaN NRs before planar selective anisotropic etching (b) selective anisotropic KOH wet chemical etching process to obtain a smaller diameter. Note the GaN NRs tip facets and sides and the hexagonal cavities left behind by an uprooted rod (circled in red). (c) SEM images of the GaN NRs on the GaN layer used for Kelvin probe studies, (d-f) Topographic images of broken GaN NRs after planar selective KOH etching showing the hexagonal geometry of the structure.

Raman measurements

Micro-Raman spectra were measured with a confocal Horiba LabRam HR Evolution, equipped with a Syncerity CCD detector (deep-cooled to – 60 ^{0}C, 1024 x 256 pixels). The excitation source was a Nd:YAG 532 nm laser with a power on the sample of 3.5 mW. The laser was focused on the sample with a 100x objective (MPLN100X, NA = 0.9) to a spot size of about 0.7 µm. The measurements were taken using an 1800 g mm^{-1} grating (spectral resolution: 0.5 cm^{-1}), with a 120 µm confocal hole. The typical exposure time was 10 s.

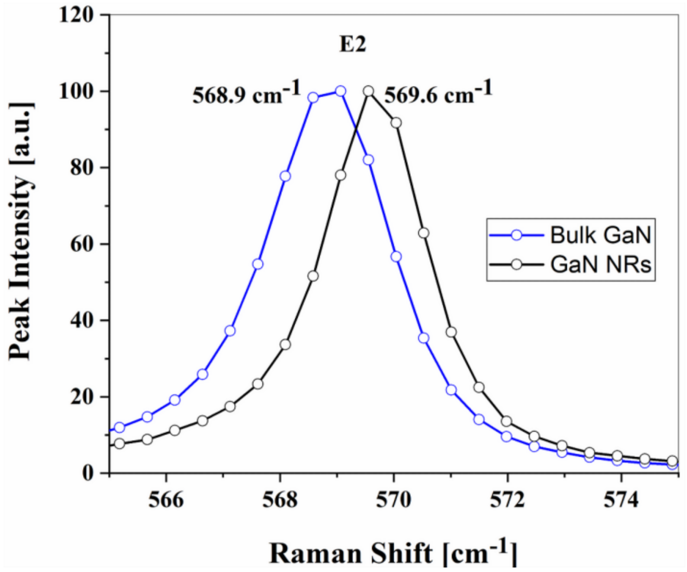

Figure S10. Raman spectra for bulk GaN and for GaN NRs samples.

Grounding the GaN samples for SPS and SPV measurements

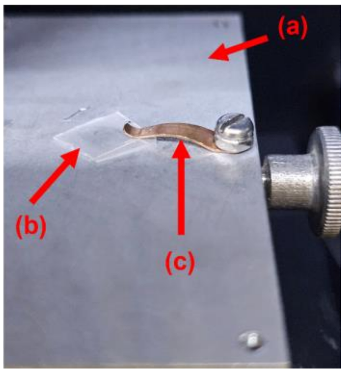

Figure S11. Picture of (a) grounded stage, connected to the top of the (b) GaN sample with (c) copper pin

References

(1) Yang, H. C.; Lin, T. Y.; Huang, M. Y.; Chen, Y. F. Optical Properties of Si-Doped GaN Films. *J Appl Phys* 1999, *86* (11), 6124–6127. https://doi.org/10.1063/1.371662.

(2) Reshchikov, M. A.; Morkọ, H. Luminescence Properties of Defects in GaN. *J Appl Phys* 2005, *97* (6). https://doi.org/10.1063/1.1868059.

(3) Majchrzak, D.; Grodzicki, M.; Ciechanowicz, P.; Rousset, J. G.; Piskorska-Hommel, E.; Hommel, D. The Influence of Oxygen and Carbon Contaminants on the Valence Band of P-GaN(0001). In *Acta Physica Polonica A*; Polish Academy of Sciences, 2019, 136, 585–588. https://doi.org/10.12693/APhysPolA.136.585.

(4) Li, D.; Sumiya, M.; Fuke, S.; Yang, D.; Que, D.; Suzuki, Y.; Fukuda, Y. Selective Etching of GaN Polar Surface in Potassium Hydroxide Solution Studied by X-Ray Photoelectron Spectroscopy. *J Appl Phys* 2001, *90* (8), 4219–4223. https://doi.org/10.1063/1.1402966.
